# The novel coronavirus outbreak in Wuhan, China

**DOI:** 10.1186/s41256-020-00135-6

**Published:** 2020-03-02

**Authors:** Hengbo Zhu, Li Wei, Ping Niu

**Affiliations:** Department of Pediatric, Renmin Hospital of Wuhan university, Wuhan, 430060 China

**Keywords:** Novel coronavirus, 2019-nCoV, COVID-19, Pneumonia, Outbreak, Wuhan, Global health

## Abstract

The novel coronavirus (2019-nCoV, or COVID-19) epidemic first broke out in Wuhan and has been spreading in whole China and the world. The numbers of new infections and deaths in Wuhan are still increasing, which have posed major public health and governance concerns. A series of mandatory actions have been taken by the municipal and provincial governments supported by the central government, such as measures to restrict travels across cities, case detection and contact tracing, quarantine, guidance and information to the public, detection kit development, etc. Challenges such as lacking effective drugs, insufficient hospital services and medical supplies, logistics, etc. have much alleviated with the solidarity of the whole society. The pandemic will definitely be ended with the continuous efforts of both national and international multi-sectoral bodies.

## Introduction

Since December 2019, a new type of coronavirus called novel coronavirus (2019-nCoV, or COVID-19) was identified in Wuhan, China. The COVID-19 has then rapidly spread to all over China and the world. It can cause symptoms including fever, difficulty in breathing, cough, and invasive lesions on both lungs of the patients [1]. It can spread to the lower respiratory tract and cause viral pneumonia. In severe cases, patients suffer from dyspnea and respiratory distress syndrome.

The pandemic has a big number of infected patients that far exceeded the equivalents of Severe Acute Respiratory Syndromes (SARS) and Middle East respiratory syndrome (MERS), though with a lower fatality rate. According to the surveillance statistics reported by the Chinese government, by February 19, 2020, the number of confirmed infection cases increased to 44,412 for Wuhan and 74,280 for whole China, with 1497 and 2009 deaths respectively. Moreover, the pandemic has caused 919 confirmed infection cases and 3 deaths globally. Therefore, Wuhan city and Hubei Province are the targets for intensive interventions. Otherwise, the spread would have been much faster to all China and the world.

Wuhan is a transportation hub of China, it is a highly dense city and has a large population of more than 14 million in 2019. The World Health Organization (WHO) had a meeting on January 30, 2020 and they declared the coronavirus outbreak from China a public health emergency of international concern. Further, there are lots of concerns and debates all over the world, indicating a need for more understanding of China’s systems in responding to the outbreak. Therefore, based on our firsthand experience of working with few of the COVID-19 cases, the purpose of this article is to have a brief report of current development, challenges, and future directions of the coronavirus outbreak in Wuhan.

## China’s progressive responses

It is recognized by the international community that China has made remarkable progress in responding effectively to the outbreak [1, 2]. What made China address the epidemic faster is its ability to finance and mobilize resources combined with its strong governance structure, efficient execution, and solidarity of the whole society. It just took 1 month for China to recognize the existence of a novel coronavirus after the first case was reported, followed with a series mandatory actions in both Wuhan and all over China. In contrast, it took more than 4 months for SARS. On December 31, 2019, delegates of the Chinese Center for Disease Control and Prevention (CDC) went to Wuhan for field investigations, and the sample of new virus was isolated and further identified as a pathogen of unexplained pneumonia on January 6, 2020. The genome-wide sequence of the virus was decoded in the next few days [3].

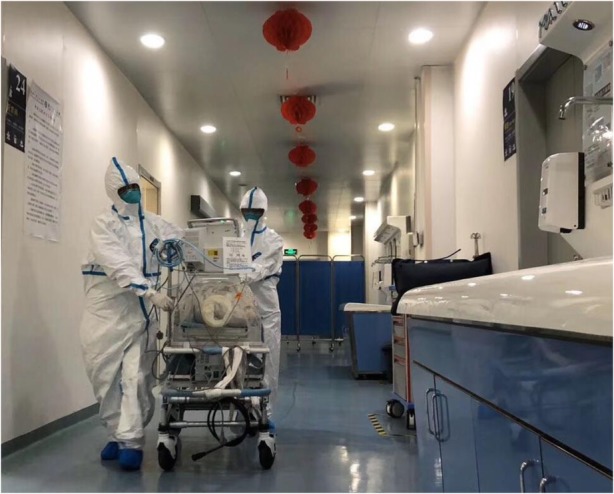



Population mobility to other cities from Wuhan was restricted on January 23, 2020, and all the inhabitants have been affected to either go out or come back to the city ever since. Vehicle transportations in Wuhan such as city buses and subways were banned and outbound transportations have been canceled (airlines, trains, and long-distance buses). In many places, the Chinese Lunar New Year Festival celebration and other gatherings were cancelled to reduce population concentration. Besides, Wuhan not only imposed a ban on overseas travels for tour purposes, but also suspended selling flight tickets and hotel-booking.

After recognizing it as an emergency epidemic on January 22, 2020, strong measures have been adopted immediately by Wuhan local authorities to characterize and control the epidemic, including isolation of suspected cases for treatment, close monitoring of contacts, epidemiological and clinical data collection from patients, and development of diagnostic and treatment procedures. More and more hospitals have been designated by the government to treat infected patients. Thousands of people have been quarantined in the new built hospitals such as Huoshenshan Hospital, Leishenshan Hospital, and Fangcang Hospital to provide care for the confirmed infection patients in Wuhan. In the meantime, patients with different severity are being treated in different hospitals. Thousands of medical professionals nationwide came to Wuhan and other cities in Hubei Province for assistance.

Many kinds of guidelines have been developed, and useful information about risk factors and preventive measures are recommended to the public by various means [4]. It is found that the COVID-19 can be transmitted through droplets, contact, aerosol, etc. [5]. A person will not be infected if he washes his hands before touching the conjunctiva. Accordingly, measures such as washing hands, wearing masks and goggles are very effective to prevent potential infections. Further, Wuhan has implemented closed management of communities. Inhabitants are not allowed to go out of their communities and they are very supportive to this regulation.

COVID-19 detection kits have been developed and the test results can be generated within 6 h, which is helpful for early diagnosis, treatment and judgment of the treatment effect. Although the number of patients with COVID-19 infections is large in Wuhan, the fatality rate is much lower (3.37%, by February 19, 2020) compared with that of SARS (11%, 2003). By February 19, 2020, 4895 people have been recovered after treatments and most of them are mild cases.

## Challenges

It is the first time for the COVID-19 to infect humans and can be transmitted from person to person [6]. The incubation period can be 2 weeks [7] and even longer. Besides, the virus can spread during the incubation period or recessive infection, which makes it difficult to identify those suspected cases without clinical symptoms for prompt control. By far, the numbers of new infections and deaths have already exceeded the equivalent numbers of cases with SARS. If the situation cannot be fully controlled in Wuhan and Hubei province, the situation may deteriorate in other places of China and the world. The government of all levels have been taking strong leadership to combat the outbreak and in recent days there is a decline trend of new cases. However, some challenges still remain and need to be addressed:

1. The large number of confirmed and suspected cases in Wuhan make people staying with them in high risk of getting infected because of the contagiousness of the new virus [8]. This is specially the case for medical professionals. By February 11, 2020, 1716 medical personnel have been infected and six of them died from all over China. Moreover, it is very difficult to identify those people without obvious symptoms, making their families in high risk of getting infected.
2. Hospitals have drastically constrained other services to meet the hospitalization needs of the outbreak in Wuhan. At the early stage, with more people getting infected and less of them recovered, medical facilities, personnel and protective supplies were increasingly insufficient. In many cases, patients cannot be quarantined and treated in time, and many medical staff cannot get fully protected. More efficient logistic services are expected to deliver donated materials from both China and the international society to medical professionals and communities. However, these are all operating problems and the situation has much alleviated with the strong leadership of government of all levels.
3. Effective drugs have yet to be developed to treat the COVID-19. Temporarily, Lopinavir/Ritonavir, Nucleoside analogues (e.g. ribavirin), Neuraminidase inhibitors (e.g. oseltamivir), Remdesivir, abidol hydrochloride, RNA synthesis inhibitors (such as Tenofovir disoproxil fumarate, TDF), anti-inflammatory drugs (such as hormones and other molecules), Traditional Chinese Medicine (TCM), such Shu Feng Jie Du Capsules and Lian Hua Qing Wen Capsule, are being used as treatment options for COVID-19, but their efficacy and safety of these drugs are yet to be clinically observed in treatments [9]. Besides, chloroquine phosphate has recently been recommended by the clinical diagnostics solution (6th edition) released by the National Health Commission of China.

## Looking ahead

Wuhan has the competence and will to manage the outbreak supported by the provincial and central governments, together with the solidarity of whole China and the international community. Wuhan has made great contributions to global health. Further global cooperation is required in order to win the pandemic war from local to global. Scientists all over the world need to understand COVID-19 and discover potentially effective antiviral treatments and vaccines; epidemiologists need to conduct intensive contact investigations; It is important to promote protection knowledge for the general public [10]. Finally, Wuhan will not cease the restrictions of population mobility and transportation until the situation is fully under control. With all people working together, it is undoubtedly the COVID-19 can be treated and the pandemic is controllable. However, comprehensive actions from multi-sectoral bodies, both national and international, with supportive attitudes will be important to proceed towards the solution.

## Data Availability

Not applicable.

## Abbreviations

2019-nCoV or COVID-19: Novel coronavirus
CDC: Center for Disease Control and Prevention
MERS: Middle East respiratory syndrome
SARS: Severe Acute Respiratory Syndromes
TCM: Traditional Chinese Medicine
TDF: Tenofovir Disoproxil Fumarate
WHO: World Health Organization
